# Increased Evoked Potentials to Arousing Auditory Stimuli during Sleep: Implication for the Understanding of Dream Recall

**DOI:** 10.3389/fnhum.2017.00132

**Published:** 2017-03-21

**Authors:** Raphael Vallat, Tarek Lajnef, Jean-Baptiste Eichenlaub, Christian Berthomier, Karim Jerbi, Dominique Morlet, Perrine M. Ruby

**Affiliations:** ^1^Brain Dynamics and Cognition Team—Lyon Neuroscience Research Center (CRNL), INSERM U1028, CNRS UMR 5292, Centre Hospitalier Le Vinatier (Bat. 452)Bron, France; ^2^Lyon 1 UniversityLyon, France; ^3^LETI Lab, Sfax National Engineering School, University of SfaxSfax, Tunisia; ^4^Department of Psychology, Université de MontréalMontréal, QC, Canada; ^5^Department of Neurology, Massachusetts General Hospital, Harvard Medical SchoolBoston, MA, USA; ^6^PhysipParis, France

**Keywords:** EEG, dreaming, awakenings, arousals, sleep, oddball paradigm

## Abstract

High dream recallers (HR) show a larger brain reactivity to auditory stimuli during wakefulness and sleep as compared to low dream recallers (LR) and also more intra-sleep wakefulness (ISW), but no other modification of the sleep macrostructure. To further understand the possible causal link between brain responses, ISW and dream recall, we investigated the sleep microstructure of HR and LR, and tested whether the amplitude of auditory evoked potentials (AEPs) was predictive of arousing reactions during sleep. Participants (18 HR, 18 LR) were presented with sounds during a whole night of sleep in the lab and polysomnographic data were recorded. Sleep microstructure (arousals, rapid eye movements (REMs), muscle twitches (MTs), spindles, KCs) was assessed using visual, semi-automatic and automatic validated methods. AEPs to arousing (awakenings or arousals) and non-arousing stimuli were subsequently computed. No between-group difference in the microstructure of sleep was found. In N2 sleep, auditory arousing stimuli elicited a larger parieto-occipital positivity and an increased late frontal negativity as compared to non-arousing stimuli. As compared to LR, HR showed more arousing stimuli and more long awakenings, regardless of the sleep stage but did not show more numerous or longer arousals. These results suggest that the amplitude of the brain response to stimuli during sleep determine subsequent awakening and that awakening duration (and not arousal) is the critical parameter for dream recall. Notably, our results led us to propose that the minimum necessary duration of an awakening during sleep for a successful encoding of dreams into long-term memory is approximately 2 min.

## Introduction

Nearly everyone has awakened once with a dream in mind. The dream recall frequency however can vary substantially between individuals and even within one person from time to time. For more than a century, researchers have investigated whether some psychological parameters could explain dream recall frequency variability (for a review see Ruby, 2011), but it’s only recently that physiological parameters have been considered. Using Electroencephalogram (EEG) and PET we found neurophysiological differences between high dream-recallers (HR) and low dream-recallers (LR) during both sleep and wakefulness (Ruby et al., 2013b; Eichenlaub et al., 2014a,b). Notably, during wakefulness, in response to auditory novel stimuli, the attention-orienting brain response (P3a) and a late parietal component were found to be larger in HR than in LR. During sleep, between-group differences were also observed for auditory evoked potentials (AEPs) to the same stimuli, at the latency of the P3a in N2 and at later latencies during all sleep stages. Finally, at the behavioral level HR showed more intra-sleep wakefulness (ISW) than LR (~15 min more on average, see Eichenlaub et al., 2014a).

Taken together, these results suggest a causal link between neurophysiological responses to auditory stimuli and ISW. We proposed that this link could be subserved by the amplitude of the brain response to external stimuli (Eichenlaub et al., 2014a), and hypothesized that large neurophysiological responses to sounds during sleep are responsible for subsequent awakenings. Indeed, Bastuji et al. (2008) demonstrated that during sleep (N2 and rapid eye movements (REMs)), the amplitude of a late parietal positive component (450–650 ms) in response to painful stimulation was significantly enhanced in trials that were followed by an arousing reaction (be it a short arousal of 3–15 s or an awakening lasting more than 15 s). The authors concluded that laser-evoked response to nociceptive stimuli during sleep predicted subsequent arousing reactions. However, such results remain to be demonstrated for auditory stimuli. This hypothesis was not tested in our previous study because of a too small number of sounds followed by an awakening which prevented us from computing reliable AEPs (Eichenlaub et al., 2014a). Awakenings (lasting at least 15 s by definition) are indeed far less frequent for auditory stimuli than for painful stimuli (Bastuji et al., 2008). Nonetheless, considering that arousals (short awakenings lasting less than 15 s) are also far more frequent than full awakenings (twice more numerous in Bastuji et al.’s (2008) study), we hoped that considering arousals in addition to awakenings would allow us to investigate event-related potentials (ERPs) to auditory arousing stimuli (which are far more frequent than painful stimuli during sleep in everyday life).

Mechanisms responsible for arousing reactions are important to consider regarding dream recall since the arousal-retrieval model postulates that ISW is the enabling factor for dreams to be encoded in long term memory (Koulack and Goodenough, 1976). Previous results from our group and other teams (Schredl et al., 2003; De Gennaro et al., 2010) argue in favor of this hypothesis but several parameters remain to be investigated to clarify and strengthen our understanding of the link between ISW and dream recall. Notably, sleep macro and microstructure need to be exhaustively investigated to identify or exclude the involvement of other sleep parameters in dream recall, and to specify the characteristics of awakenings associated with a higher dream recall frequency (e.g., distribution in the sleep cycle, duration and alpha frequency).

The goals of the present study were to further investigate the hypothesis of a causal link between AEPs and subsequent arousing reactions (Eichenlaub et al., 2014a) and the hypothesis of a causal link between ISW and dream recall (Koulack and Goodenough, 1976; Eichenlaub et al., 2014a). To this purpose, we first scored arousals in data previously acquired and reported in Eichenlaub et al. (2014a), to have a full assessment of the arousing reactions in the subjects’ sleep. Thanks to this scoring, AEPs to arousing stimuli (i.e., stimuli followed by an arousal or a full awakening in the first 15 s post stimulus) and to non-arousing stimuli were then computed. Second, several parameters of the sleep macrostructure were computed such as the distribution of awakenings and the number of stage shifts across the sleep cycle, spectral power of the delta rhythm during the first sleep cycle, frequency and spectral power of the alpha rhythm during intra-sleep and before-sleep wakefulness (BSW). Finally, as many previous results justified to investigate sleep microstructural components regarding dream recall, a thorough analysis of the sleep microstructure of HR and LR was conducted. Sleep spindles and the alpha frequency during wakefulness have been related to memory abilities (Klimesch, 1997; Ulrich, 2016), the REMs of REM sleep to dream content (Dement and Kleitman, 1957; Roffwarg et al., 1962; Molinari and Foulkes, 1969), K-complexes (KCs) to sleep stability (Bastien et al., 2000; Colrain, 2005; Halász, 2005) and muscles twitches may be considered as the physiological expression of oneiric behaviors thought to be an acting out of dreams (Wolpert, 1960; Sastre and Jouvet, 1979). Each of these parameters was computed using either visual (arousals), semi-automatic (REMs, muscle twitches (MTs), KCs) and automatic validated methods (alpha, spindles). Such a systematic analysis of macrostructural and microstructural sleep parameters have scarcely been reported in healthy subjects, especially using automatic methods, and never among HR and LR.

## Materials and Methods

We re-analyzed data presented in previous articles (Eichenlaub et al., 2012, 2014a; Ruby et al., 2013a,b).

### Subjects

Eighteen HR (age = 22.7 ± 0.6 years old; dream recall = 4.4 ± 0.25 mornings per week) and 18 LR (age = 22.4 ± 0.9 years old; dream recall = 0.25 ± 0.02 morning per week) were selected out of 1000 participants who completed an online questionnaire on their sleep and dream habits (importantly, they were unaware that dream recall frequency was an inclusion criterion). Participants were subsequently contacted by telephone and selected as HR or LR upon confirming dream recall on >3 mornings per week or <2 mornings per month respectively. Gender, age, habitual sleep duration, habitual sleep time, education level and the size of the place of residence of the subjects were controlled and did not differ between the two groups. The local ethics committee (Centre Leon Bérard, Lyon) approved this study, and subjects provided written, informed consent in accordance with the Declaration of Helsinki. The subjects were paid for their participation. For more details concerning inclusion criteria refer to Eichenlaub et al. (2014a).

### Stimuli

During both sleep and pre-sleep wakefulness subjects were presented with two different first names included as novels in an oddball novelty paradigm. The first names consisted of the first name of the subject (novel 1) and an unfamiliar first name (novel 2). They were normalized for duration and amplitude and presented with a probability of occurrence of 0.02 each. Responses to frequent standard tones (*p* = 0.82) and rare deviant tones (*p* = 0.14) were not considered in this study. Stimulus onset asynchrony was set at 650 ms, except for the standard following a novel, which appeared 1260 ms after the novel onset, whatever the duration of the novel. Stimuli were presented in a pseudo-randomized order (Eichenlaub et al., 2012), binaurally and at 50 dB above the hearing threshold of the subject.

### Electrophysiological Recordings

EEG data were recorded from 21-Ag/AgCl electrodes placed according to the extended International 10–20 System. The electro-oculogram (EOG) was recorded from two electrodes placed on the supraorbital ridge of the left eye and on the infraorbital ridge of the right eye. Muscle activity electromyogram (EMG) was recorded from two electrodes attached to the chin. Polysomnographic data (EEG, EMG, EOG) were continuously recorded using a BrainAmp system (Brain Products GmbH, Germany) with an amplification gain of 12,500, a high-pass filter of 0.1 Hz, and a sampling rate of 1000 Hz.

### Procedure

Subjects slept at night in a bed in the lab while polysomnographic data were acquired. Stimuli were presented through earphones during the whole night, thus ensuring constancy of stimulus input throughout the night despite head movements (Cote et al., 1999). When possible, subjects were awakened in the morning by the experimenters after 5–10 min of REM sleep and were subsequently asked to report any dreams or sleep mentation. For more details see Eichenlaub et al. (2014a).

### Data Analysis

#### Sleep Macrostructure

Polysomnographic data have been scored according to the standard guidelines (Iber et al., 2007; Silber et al., 2007) using 30-s epochs to identify the sleep macrostructure of the subjects. Scoring was done both by an automatic software (ASEEGA version 3.30.14, Physip, France, Berthomier et al., 2007) and by an expert scorer (JBE; for more details see Eichenlaub et al., 2014a). The epoch-by-epoch comparison between JBE and ASEEGA shown 83.6% of agreement with a Cohen’s kappa coefficient of 0.77. All further analyses in the present study are based solely on the visually-scored hypnograms. For each group, the following sleep parameters were measured.

##### Awakenings

Total number of awakenings over the sleep period time; Awakening index, defined as the number of awakenings per hour of sleep, computed for the total sleep time (TST), N1, N2, N3 and REM sleep; Awakening duration, defined as the average duration of awakenings; Awakening type, defined as the percentage of long and short awakenings (0–1 min, 1–5 min and 5–30 min, as in Goldenberg et al., 1981).

##### Stage shifts

Total number of stage shifts over the sleep period time (includes transitions from and to W, N1, N2, N3 and REM).

#### Sleep Microstructure

##### Arousals

Arousals were visually scored according to the ASDA 92 criteria (American Sleep Disorders Association, 1992). They were defined as any shift in the EEG frequency to alpha or theta for at least 3 s irrespective of changes in submental EMG during NREM sleep but accompanied by a at least 3 s increase in EMG amplitude during REM sleep. Typically, arousals last between 3 s and 15 s (see Supplementary Figure S1 for an example of arousal). However, since arousal scoring is independent from the scoring of sleep macrostructure, arousals may last more than 15 s (but not more than 30 s without being considered as awakenings according to the standard guidelines (if they start on one page and continue on the next one). Moreover, the ASDA rules also state that an arousal has to be preceded by at least 10 s of continuous sleep which means that a minimum of 10 s of intervening sleep is necessary between two arousals. Arousal were scored by RV and PR. RV scored arousal first, PR reviewed the scoring realized by RV and proposed modifications, then RV checked the propositions and in case of disagreement a consensus was reached after discussion. The parameters that were considered in the present study are: (1) the total number of arousals over the sleep period time; (2) the arousal index (AI), defined as the number of arousals per hour of sleep, computed for TST, N1, N2, N3, REM as well as for epochs scored as indeterminate (Inds) and movements (Mvts); and (3) the arousal duration, defined as the average duration of arousals.

##### Rapid eye movements

REMs are a core feature of REM sleep (Iber et al., 2007; Silber et al., 2007). In order to assess if there is any difference in the amount of REMs during REM sleep between HR and LR, we developed a semi-automatic detection of saccades in the EOG (see Supplementary Figure S2). We used independent component analysis to extract EOG-components free from artifacts from the original polysomnographic data. Then for each subject, two components showing eye movements were visually selected and subsequently analyzed with the following method. The signal was smoothed using a moving average with a window of 100 ms. The first derivative of the signal was computed and converted into absolute value. A 40 ms step was chosen for the derivative since most of naturally occurring human saccades have magnitudes of 15° or less and last thus no more than 30–40 ms (the maximum velocity of a saccade is above 500°/s, see Bahill et al., 1975). We then applied an arbitrary threshold (mean + 4 standard deviations (SD)) to allow for an optimal detection of REMs. Finally, the quality of the detection was visually checked on a subpart of the data of several subjects. The total number of REMs was defined as the sum of non-concomitant REMs detected in the two components during REM sleep.

##### Muscle twitches

REM sleep is characterized by a flat muscle tone (atonia) and MTs which can be identified using electromyographic recordings with electrodes on the chin (Iber et al., 2007; Silber et al., 2007). To compare MTs between HR and LR, a semi-automatic analysis of the EMG was developed (see Supplementary Figure S3). First, a notch filter at 50 Hz was applied on the EMG signal. Second, the envelope of the signal was computed using standard Hilbert transform (frequency band: 60–450 Hz; Vidal et al., 2012). The obtained envelope was normalized and smoothed using a moving average with a 500-ms window. As for REMs, a threshold (3.25 SD) was empirically chosen to obtain an optimal detection of MTs. Each supra-threshold clusters with a duration superior to 100 ms were scored as MTs. Finally, the quality of the detection was visually checked on a subpart of the data of several subjects. Three subjects were excluded from the analysis because of a too noisy EMG signal. We assessed the total number of REMs and MTs in REM sleep and the density of REMs/MTs, defined as the average number of REMs/MTs per minute for the total REM sleep duration.

##### Sleep spindles

A sleep spindle is a train of distinct 11–16 Hz waves, predominant over central EEG derivations and lasting more than 0.5 s (Iber et al., 2007; Silber et al., 2007). An automatic data-driven method was used to detect sleep spindles (ASEEGA). This iterative approach uses recording-specific automatic thresholds, based on EEG power ratios in frequency bands. The detection was performed on CzPz channel, both raw EEG data and sigma-filtered EEG were used in the analysis (for more details refer to Dang-Vu et al., 2015).

##### K-complexes

K-complexes (KCs) are defined by the AASM (Iber et al., 2007; Silber et al., 2007) as *“a well delineated negative sharp wave immediately followed by a positive component with a total duration ≥0.5 s, typically maximal at frontal electrodes”*. KCs were semi-automatically detected using an open-access validated method (Lajnef et al., 2015, 2017) which is based on a combination of the tunable Q-factor wavelet transform and a morphological component analysis. This approach requires an initial calibration step where a small subset of the data is visually scored for KCs and then used to derive an optimal threshold. Once the training is achieved, the algorithm runs on the entire dataset to automatically detect KCs. For both KCs and spindles, the outcome measures were the total number of KCs/spindles detected and the density which is the number per min of N2 sleep.

#### Spectral and Frequency Analysis

##### Delta power

EEG delta spectral power in the first sleep cycle is a marker of homeostatic sleep pressure (Achermann et al., 1993; Huber et al., 2004). Calculation of the normalized spectral power of CzPz in the delta band (0.1–4 Hz) was performed using a fast Fourier transform with Hanning window for consecutive 30-s epochs after automatic artifact rejection (ASEEGA).

##### Alpha power and predominant frequency

A specific analysis of the alpha band (8–12 Hz) was performed after automatic artifact rejection. For each 30 s epoch of the EEG CzPz signal, the alpha normalized power and the mean of the instantaneous frequency weighted by the alpha power (Berthomier, 1983) were computed (ASEEGA). We assessed the predominant frequency and normalized power of alpha band for intra-sleep wakefulness (ISW) and BSW epochs and the normalized power of delta band in the first sleep cycle.

For both delta and alpha power, values are reported in percent after band-wise normalization in five frequency bands (delta = 0.1–4 Hz, theta = 4–8 Hz, alpha = 8–12 Hz, beta = 12–16 Hz, sigma = 16–50 Hz) in order to avoid low frequency artifact and powerline artifacts. Hence, for each 30-s epoch, the sum of normalized values in the five bands is equal to 100.

#### Event-Related Potentials Analysis

AEPs were analyzed using Elan pack software (Aguera et al., 2011) and Matlab (Mathworks). AEPs elicited by first names were averaged over an epoch of 1100 ms, including a prestimulus period of 100 ms. Prior to averaging, trials were automatically excluded if the overall electrophysiological signal amplitude exceeded 400 μV during N2. Epoch were baseline corrected according to the pre-stimulus period and a 30-Hz low-pass butterworth (order 3) digital filter was applied to averaged responses. Arousing reactions were considered as stimulus-related if occurring within 15 s after stimulus onset (American Sleep Disorders Association, 1992).

#### Statistical Analysis

Between-group comparisons of the sleep characteristics were achieved using Student *t*-tests (two-tailed, level of significance, *p* < 0.05). In addition, a nested two-way ANOVA tested the sleep stage (four levels: N1, N2, N3, REM sleep) and the group (two levels: HR, LR) effects on the parameters of the macrostructure (number and duration of awakenings) and microstructure of sleep (number and duration of arousals). *Post hoc* analyses (*t*-tests) were used in case of significance.

The analysis of the AEPs was performed using non-parametric tests at each sampling point and at each *a priori* chosen electrode (Fp1, Fp2, F3, Fz, F4, C3, Cz, C4, P3, Pz, P4, O1, O2) for grand averaged responses. We used Wilcoxon matched rank sign tests to compare AEPs between conditions (arousing vs. non-arousing; two-tailed, *p* < 0.05). To take into account the issue of multiple comparisons, we chose to apply both spatial limitations based on previous results (according to the results of Bastuji et al. (2008) and Eichenlaub et al. (2014a), we expected condition effects for the contrast arousing stimuli vs. non-arousing stimuli on AEPs at frontal and parieto-occipital electrodes), i.e., to consider only a limited amount of electrodes, and temporal and spatial constraints to decrease the chances of false positive, i.e., considering a difference significant only if more than 15 consecutive samples (15 ms) were significantly different for at least two adjacent electrodes (Guthrie and Buchwald, 1991). This method has the advantage of taking spatial and temporal priors into account and is classically used in electrophysiology to correct for multiple comparisons (e.g., Caclin et al., 2008; Bidet-Caulet et al., 2012; Eichenlaub et al., 2014a).

## Results

### Sleep Parameters

Table 1 shows the main sleep parameters of the experimental night in the lab for the two groups reported in Eichenlaub et al.’s (2014a) study (reproduced with permission). HR showed more ISW (wake after sleep onset, WASO) in average than LR and consequently a shorter TST. Except for these parameters, no other between groups difference was observed in the sleep architecture (notably in the proportion and latency of each sleep stage).

**Table 1:** **Mean ± SEM of the main sleep parameters obtained in the original study**.

Sleep parameters	High-recallers	Low-recallers	Standard
TIB (min)	449 ± 10	479 ± 15	*390–510*
SPT (min)	428 ± 11	449 ± 11	
WASO (min)	30 ± 4*	14 ± 5	*20–30*
TST (min)	398 ± 11*	435 ± 12	
Sleep efficiency (%)	89 ± 1.4	91 ± 1.8	*80–90*
Sleep stage, % of TST
N1 (%)	4 ± 0.6	2 ± 0.6	*5–10*
N2 (%)	39 ± 1.7	41 ± 2.1	*40–55*
N3 (%)	36 ± 1.5	36 ± 2.3	*25–30*
REM sleep (%)	21 ± 1.2	21 ± 1.1	*20–25*
N2 latency from lights out (min)	19 ± 2.6	29 ± 6.2	*20–30*
N3 latency from lights out (min)	21 ± 3.3	32 ± 6.3	
REM latency from lights out (min)	120 ± 13.2	133 ± 16.4	
N3 latency from N2 (min)	5 ± 1.8	5 ± 1.5	
REM latency from N2 (min)	104 ± 11.9	106 ± 12.9	*60–120*
WASO (%)	7 ± 0.9*	3 ± 1.1	*5*
Movements (%)	4 ± 0.5	4 ± 0.5	
Indeterminate (%)	10 ± 1.4	10 ± 0.9	

Table 1 is reproduced and modified from Eichenlaub et al. (2014a) with permission of the authors. Only values obtained with visual scoring (JBE) are reported. TIB refers to time in bed; SPT, sleep period time; WASO, wakefulness after sleep onset; TST, total sleep time; REM, rapid eye movement sleep; min, minutes. In comparison with the standard values presented in the last column (Hirshkowitz, 2004), the quality of sleep was generally preserved for the 36 subjects. *p < 0.05.

Table 2 presents the new parameters of the macrostructure (notably the distribution of awakenings across the sleep cycle and the proportion of long and short awakenings) and several parameters of the microstructure of sleep that have been investigated thanks to the re-analysis of the data.

**Table 2:** **Mean ± SEM of supplementary macrostructural and microstructural sleep parameters calculated in this study**.

Sleep parameters	High-recallers	Low-recallers	Standard
**Sleep macrostructure**
Awakenings, no.	17.5 ± 2.1	12.1 ± 2.9	9.6 (Hirshkowitz, 2004)
Awakenings, duration (min)	1.9 ± 0.2**	0.95 ± 0.1	1.4 (Benoit et al., 1981)
Awakenings index, no. per hour	3.2 ± 0.4	2.2 ± 0.6	4.2 (Wamsley et al., 2012)
N1	27.3 ± 4.8	34.6 ± 7	
N2	3.3 ± 0.7	1.8 ± 0.7	
N3	0.9 ± 0.2	1.1 ± 0.3	
REM	3.6 ± 1.5	1.0 ± 0.3	
Awakenings duration (%)
0–1 min	66.1 ± 3.2**	82.9 ± 3.3	87 (Goldenberg et al., 1981)
1–5 min	24.6 ± 2.1*	16.1 ± 3.3	11 (Goldenberg et al., 1981)
5–30 min	9.4 ± 2.5**	1.0 ± 0.6	3 (Goldenberg et al., 1981)
Number of stage shifts	63.6 ± 4.2	71.6 ± 7.2	47 (Hirshkowitz, 2004)
Alpha power (%), in B.S.W	32 ± 4.6	30.5 ± 3.9	
Alpha power (%), in I.S.W	21 ± 3.1	24.4 ± 2.5	
Alpha frequency (Hz), in B.S.W	9.88 ± 0.1	9.86 ± 0.1	
Alpha frequency (Hz), in I.S.W	9.64 ± 0.1	9.65 ± 0.1	
Delta power (%), in first sleep cycle	79.7 ± 1.7	80.8 ± 1.6	
Arousing stimuli (%)	2.9 ± 0.3**	1.6 ± 0.2	
Arousing, latency after stim. onset	5.3 ± 0.2	5.8 ± 0.3	
**Sleep microstructure**
Arousals, no.	76.3 ± 8.5	61.2 ± 7.2	83 (Bonnet and Arand, 2007)
Arousals, duration (sec)	10.2 ± 0.3	11.2 ± 0.4	
Arousal index	11.1 ± 1	8.3 ± 1	10.8 (Bonnet and Arand, 2007)
Spindles, no. per min. of N2	3.5 ± 0.3	3.5 ± 0.4	2.1 (Wamsley et al., 2012)
K-complex density, no. per min. of N2	2.0 ± 0.2	2.5 ± 0.2	1–3 (Halász, 2005)
REMs, no. per min of REM sleep	10.9 ± 0.3	10.3 ± 0.3	3.7 (Andrillon et al., 2015)
MTs, no. per min of REM sleep	0.8 ± 0.1	0.8 ± 0.1	

REMs refers to rapid eye movements; ISW, intra-sleep wakefulness; BSW, before-sleep wakefulness; TST, total sleep time; Normalized spectral power (alpha and delta) is relative to the power in the other frequency bands; MT, muscle twitches; min, minute; no, number. Sleep onset was considered as the first epoch of N2 for the calculation of intra-sleep awakenings. Sleep onset was considered as the first epoch of N1 for arousals. Supplementary Table S1 reports awakening parameters using the first epoch of N1 as sleep onset. Last column represents standard values. One-way and two-way ANOVA for independent samples (High-recallers vs. Low-recallers) are presented: *p < 0.05, **p < 0.01.

#### Awakenings

**Figure 1:**
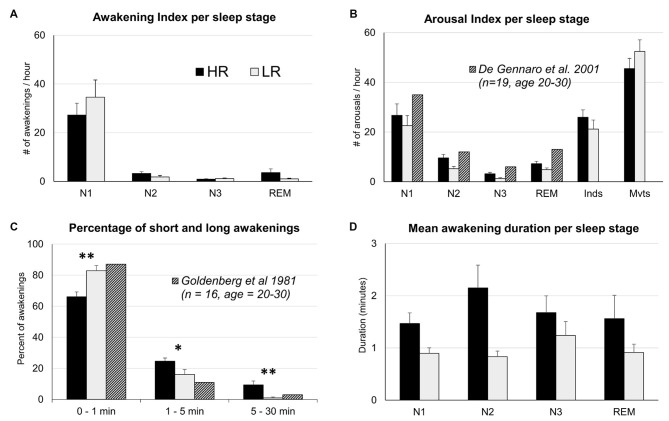
**Means and SEM of sleep parameters for High and Low dream recallers (HR and LR). (A)** Awakening index per sleep stage (number of awakenings per hour in each sleep stage). **(B)** Arousal index (AI) per sleep stage (number of arousals per hour in each sleep stage). **(C)** Percentage of short and long awakenings. **(D)** Mean duration of awakenings per sleep stages. Hatched columns represent standards values. *p < 0.05, **p < 0.01.

A two-way ANOVA yielded a significant sleep stage effect on the awakening index (*F*_(1,3)_ = 47.0, *p* < 0.000), no significant group effect and no group × sleep stage interaction (Figure 1A). *Post hoc* tests revealed that the awakening index in N1 was significantly higher than in all other sleep stages (*p* < 0.000 for all the comparisons between N1 and another sleep stage) and that it was also higher in N2 than in N3 (*p* = 0.003). Regarding the duration of awakenings, a two-way ANOVA showed a group effect (*F*_(1,3)_ = 13.6, *p* < 0.001) but no sleep stage effect or interaction group × sleep stage (Figure 1D). After classifying awakenings into three categories according to their duration i.e., 0–1 min, 1–5 min and 5–30 min (Goldenberg et al., 1981), we observed a significant interaction group × duration (*F*_(1,2)_ = 14.75, *p* < 0.001; Figure 1C). *Post hoc* comparisons revealed that HR showed less short awakenings lasting less than 1 min (66.1 ± 3% in HR vs. 82.9 ± 3% in LR, *p* < 0.001) but more long awakenings lasting more than 1 min than LR (1–5 min, 24.6 ± 2% in HR vs. 16.1 ± 3% in LR, *p* = 0.03; 5–30 min, 9.4 ± 2.5% in HR vs. 1 ± 0.6% in LR, *p* = 0.003).

#### Arousals

The total number of arousals was not significantly different between the two groups (*p* = 0.19). A two-way ANOVA on the AI showed a significant sleep stage effect (*F*_(1,5)_ = 88, *p* < 0.001), no group effect (a tendency towards a larger AI in HR than in LR was observed regardless of the sleep stages, *p* = 0.07) and no group × sleep stage interaction (Table 2). The distribution of arousals across sleep stages is in accordance with previous studies (De Gennaro et al., 2001) even if AI was slightly lower than expected in all sleep stages (Figure 1B). In De Gennaro et al.’s (2001) study, the categories indeterminate (Inds) and movements (Mvts) were not considered in the sleep scoring whereas in the present study the majority of arousals were found in epochs scored as Inds or Mvts. This could explain the lower AI in each sleep stage (N1, N2, N3 and REM sleep) found in our study. The AI did not correlate significantly with any other sleep parameters, including the awakening index (Pearson’s *r* = 0.24, *N* = 36, *p* = 0.15, two-tailed). A two-way ANOVA on arousals duration showed a significant sleep stage effect (*F*_(1,5)_ = 35, *p* < 0.000), but no group effect nor interaction group × sleep stage. Arousals were significantly longer in epochs scored as Mvts than all other epochs (N1, 9.7 ± 0.6 s, N2, 8.5 ± 0.4, N3, 9.5 ± 0.7, REM, 9.3 ± 0.5, Inds, 9.1 ± 0.3, Mvts, 16.4 ± 2.0, *p* < 0.000 for all pairwise comparisons between Mvts and the other stages).

#### Arousing Stimuli

In average (*N* = 36), 2.2 ± 0.2% of auditory stimuli (first names) that occurred during either N2, N3 or REM sleep were associated with an arousing reaction (be it an arousal or an awakening) in the following 15 s (see Supplementary Figure S1 for examples of arousing and non arousing stimuli). A two-way ANOVA showed a sleep stage effect (*F*_(1,2)_ = 13.2, *p* < 0.001), a group effect (*F*_(1,2)_ = 13.1, *p* = 0.001) and no interaction effect on the rate of arousing stimuli. *Post hoc* comparisons revealed that there was a higher rate of arousing stimuli in N2 and REM as compared to N3 sleep (N2, 3.1 ± 0.4%; REM, 2.4 ± 0.3%; N3, 1.4 ± 0.2%; *p* < 0.000 for the two comparisons), but no differences between N2 and REM. HR showed a higher proportion of arousing reactions than LR (HR, 2.9 ± 0.3%, LR, 1.6 ± 0.2%, *p* = 0.001), whatever the sleep stage (no significant interaction was observed). The latency between the stimulus onset and the beginning of the arousing reaction was 5.6 ± 0.2 s in average. A two-way analysis of variance revealed a significant sleep stage effect on this delay (*F*_(1,2)_ = 3.7, *p* = 0.03), no group effect or interaction group × sleep stage. *Post hoc* tests showed that arousing reactions were more delayed in REM sleep (6.5 ± 0.4 s) than in N2 (5.0 ± 0.2 s) and N3 (5.2 ± 0.3 s) (*p* < 0.01 and *p* < 0.05 respectively). Most arousing stimuli were associated with short arousing reactions (75.6 ± 2.5% of the arousing stimuli were followed by an arousal, the remaining 24.4% were followed by an awakening). A two-way ANOVA yielded a significant sleep stage effect on the type of arousing reaction (*F*_(1,2)_ = 23.7, *p* < 0.000) but no group effect or interaction group × sleep stage. Arousing reaction in N3 were less often short arousals (50.8 ± 6.1%) than in N2 (89.1 ± 2.7%) or REM sleep (87.8 ± 3.1%; *p* < 0.000 for the two tests).

#### Other Microstructural Parameters

Number of stage shifts, sleep spindles density, KCs density, REMs density and MTs density were not significantly different between the two groups (*p* = 0.35, *p* = 0.98, *p* = 0.16, *p* = 0.25 and *p* = 0.74 respectively; see Table 2 for details).

#### Spectral Power and Predominant Frequency

Two-way ANOVAs yielded that the alpha predominant frequency and spectral power were significantly decreased during ISW as compared to BSW (*Frequency*, *F*_(1,1)_ = 32.1, *p* < 0.000. *Power*, *F*_(1,1)_ = 22.2, *p* < 0.000). For both parameters there were no group effect or interaction between group and time of the night. Moreover, delta power in the first sleep cycle was not significantly different between the two groups.

### Auditory Evoked Potentials

#### Arousing Stimuli after Artifact Rejection

N2—3.0 ± 0.4% of auditory stimuli were associated with an arousing reaction within 15 s (13.6 ± 1.8 stimuli in average per subject). Arousals prevailed (89.5%) over awakenings (10.5%). The mean latency between stimulus onset and arousing reactions was 4.5 s in N2.

REM sleep —2.2 ± 0.3% of auditory stimuli were associated with an arousing reaction within 15 s (4.2 ± 0.5 stimuli in average per subject). Arousals prevailed (87.7%) over awakenings (12.3%) The mean latency between stimulus onset and arousing reactions was 6.2 s in REM sleep.

As expected for auditory stimuli (Bastuji et al., 2008; Bastuji and Lavigne, 2016), a small percentage of stimuli led to an arousing reaction. For the evoked potential analysis, to avoid a poor signal-to-noise ratio, we retained for further analysis subjects with at least 10 arousing stimuli in the same sleep stage. These constraints restricted the analysis to the most represented sleep stage i.e., N2, for which 19 subjects (12 HR and 7 LR) showed more than 10 arousing stimuli after artifact rejection.

#### AEPs to Arousing vs. Non-Arousing Stimuli in N2

**Figure 2:**
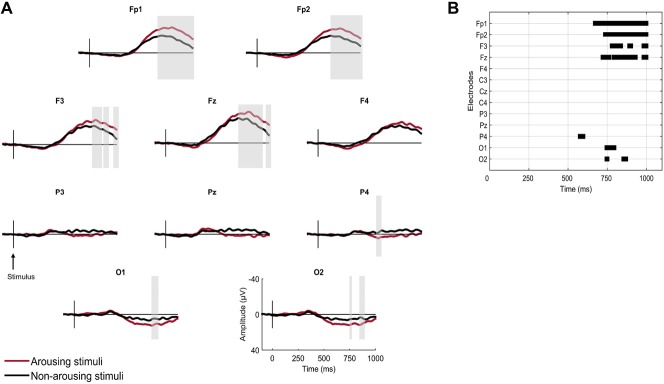
**Brain responses to first names according to the presence or absence of an arousing reaction following the stimulus in N2 sleep. (A)** Grand averaged responses. Gray vertical bars highlight significant differences (sample-by-sample Wilcoxon test, *p* < 0.05 for more than 15 ms). **(B)** Statistical significance of sample-by-sample Wilcoxon test (*p* < 0.05 for more than 15 ms) performed at 10 electrodes in the post-stimulus period.

Grand averaged responses to arousing and non-arousing stimuli in N2 are displayed in Figure 2. A sample of non-arousing stimuli was randomly selected so that the number of averaged stimuli was equivalent for the arousing and non-arousing conditions. Significant differences between the two conditions in N2 were found at frontal and occipital topographies. The amplitudes of a late positive and a late negative (peaking at 800–1000 ms) components were significantly enhanced for arousing stimuli at occipital electrodes (O1 and O2) and frontal electrodes respectively (FP1, FP2, Fz, F3).

#### AEPs to Arousing vs. Non-Arousing Stimuli in N2 as a Function of the Delay Between Stimuli and Arousing Reactions

**Figure 3:**
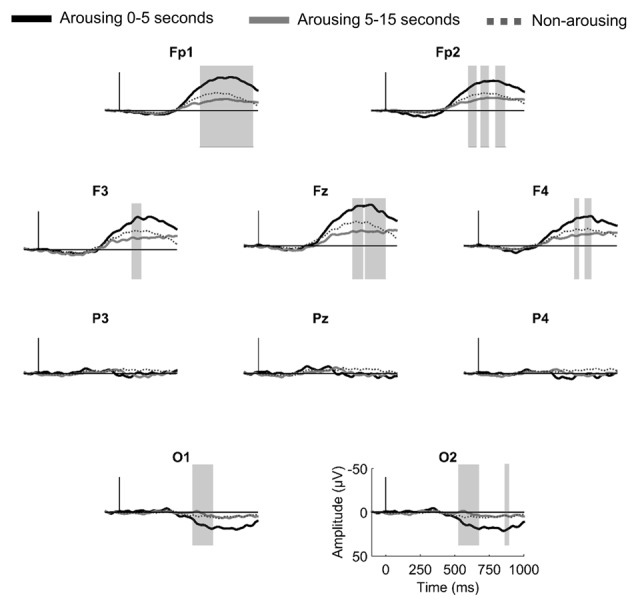
**Brain responses to first names leading to a rapid arousing reaction (in the 5 s post stimulus, black thick line), to a delayed arousing reaction (between 5 s and 15 s post-stimulus, gray thick line) and to no arousing reactions (dotted line) in N2 sleep.** Gray vertical bars highlight significant differences between rapid and delayed arousing reactions (sample-by-sample Kruskal-Wallis test, *p* < 0.05 for more than 15 ms).

According to the hypothesis that the amplitude of the AEPs to stimuli presented during sleep is related to the subsequent reaction of the sleeper, one would expect that the shorter the delay between the stimulus and the arousing reaction, the larger the amplitude of the evoked response to the stimulus. To test this hypothesis, we compared AEPs to stimuli quickly followed by an arousing reaction (arousing reaction in the 5 s after stimulus onset) and to stimuli followed by a late arousing reaction (5–15 s after stimulus onset). For this analysis paired statistics were not possible because of an insufficient number of subjects having more than 10 events in both conditions. Instead, we used the Kruskal-Wallis test to compare nine subjects with more than 10 events within 0–5 s post-stimuli (18.5 ± 2.1 events in average) and 10 subjects with more than 10 events within 5–15 s post-stimuli (16.7 ± 1.7 events in average). Grand averaged responses are displayed in Figure 3. At occipital electrodes, the amplitude of a late parietal positivity was significantly enhanced when the arousing reaction occurred within the first 5 s as compared to the last 10 s. A comparable effect was observed at frontal electrodes for the amplitude of a large negative wave peaking between 800 ms and 1000 ms.

## Discussion

In this study, we further investigated the hypothesis of a causal link between ERPs to auditory stimuli and subsequent arousing reactions (Eichenlaub et al., 2014a), and the hypothesis of a causal link between ISW and dream recall (Koulack and Goodenough, 1976; Eichenlaub et al., 2014a). The reanalysis of EEG data (Eichenlaub et al., 2012, 2014a; Ruby et al., 2013a,b) enabled us to characterize more precisely the sleep macro and microstructure of HR and LR thanks to visual, semi-automatic and validated automatic methods. For intra-sleep awakenings no interaction between group and sleep stages was found (be it for the awakening index or for awakenings duration) showing that awakenings were longer in HR than in LR whatever the sleep stage. We also observed significantly more long awakenings (longer than 1 min) and less short ones in HR than in LR. Regarding the alpha predominant frequency and alpha power during ISW and BSW, no between-group differences were found, but the overall frequency and power of the alpha rhythm was slightly lower in ISW than in BSW. There were no between-group differences in the delta power during the first sleep cycle or in the number of stage shifts during SPT. Moreover, no between-group difference was found for the parameters of the sleep microstructure considered, namely arousals (density, duration and distribution), sleep spindles (density in N2), KCs (total number and density in N2), REMs (total number and density in REM sleep), MTs (total number and density in REM sleep). Finally, thanks to the scoring of arousals, we could compute AEPs to arousing and non-arousing stimuli and reproduce the results obtained for painful stimuli showing that the amplitude of the AEPs was predictive of the subsequent arousing reaction.

### AEPs to Arousing and Non-Arousing Stimuli

The scoring of arousals in Eichenlaub et al.’s (2014a) data resulted in a great increase of the stimuli categorized as arousing. Still, auditory stimuli presented at an average intensity (50 dB above the hearing level of the subject) induced far less arousing reaction (2.2%) than did laser nociceptive stimuli (30.6% in Bastuji et al., 2008). Among all the arousing reactions, arousals largely prevailed over awakenings (three-to-one ratio). Considering both arousals and awakening improved our ability to test whether AEPs amplitude to stimuli presented during sleep was related to subsequent arousing reactions, but only in N2, the most represented sleep stage. Results revealed that auditory first names followed within 15 s by an arousing reaction elicited a larger occipital positivity and also a larger frontal negative wave than non-arousing stimuli. Similarly Bastuji et al. (2008) found that arousing laser nociceptive stimuli induced a larger late parietal positive component than non-arousing stimuli in N2 and REM sleep. We further found that the shorter (less than 5 s vs. more than 5 s) the delay between the stimulus onset and the subsequent arousing reaction, the larger the amplitude of the AEPs. These results tie convincingly the AEPs’ amplitude to subsequent arousing reaction and question the fact that arousing reaction arising between 5 s and 15 s after the stimulus onset are truly stimulus related. Our results show that arousing auditory stimuli induce arousing reaction through a cerebral mechanism similar to the one described for nociceptive stimuli i.e., an increased amplitude of the evoked response. Interestingly the topographies of the responses for the two sensory modalities are different. No frontal difference was found between ERPs to arousing and non-arousing stimuli in Bastuji et al.’s (2008) study whereas we found a large one in our study. The large amplitude of this frontal wave might have masked the parietal component that was reported in Bastuji et al.’s (2008) study on parietal electrodes. It may explain that the arousing effect appeared only on occipital electrodes in our study since they are less influenced by frontal components.

The late parieto-occipital components have traditionally been associated with complex cognitive processes, such as semantic or memory processes (Curran, 2004; Eichenlaub et al., 2012). This could reflect a high level cognitive processing of the stimuli possibly triggering a subsequent arousing reaction. The frontal component, larger for arousing auditory stimuli, is thought to reflect evoked KCs or N550 (Cote et al., 1999; Bastien et al., 2002; Bastuji et al., 2008). According to previous work, KCs have been traditionally viewed as indicative of an arousal process (for a review see Colrain, 2005). Halász (2005) also suggested that they could reflect an arousal response to exogenous stimuli, since they are often followed by arousing reactions. This view fits well with the fact that this frontal response is enhanced for auditory arousing stimuli as compared to non-arousing stimuli, but it does not fit with the fact that this component is more prominent for the auditory stimuli than for painful stimuli which are more arousing. This frontal response may rather be explained by an opposite functional role proposed for KCs. Some studies have shown that there is an increased proportion of KCs in situation of increased sleep drive and thus decreased arousability (Bastien et al., 2000, 2002; Nicholas et al., 2002; Peszka and Harsh, 2002; Colrain, 2005). These latter results are more in favor of an endogenous nature of KCs which could help maintaining sleep (Jahnke et al., 2012). This interpretation of KCs may explain that auditory stimuli are less arousing than painful ones due to their elicitation of a sleep-protective component (the frontal component) in addition to an arousing component (the parieto-occipital one).

These findings suggest that the amplitude of ERPs to stimuli presented during sleep is predictive, whatever the sensory modality, of a subsequent arousing reaction. Such a link was previously shown for somatosensory painful stimulation—i.e., potentially dangerous stimuli—but not for sounds which are much more frequent in our daily life and far less often dangerous than painful ones.

### Intra-Sleep Awakenings and Dream Recall

The increased amount of ISW in HR reported in our previous work raised several questions. If ISW is increased in HR as compared to LR, is it also true for arousals? What is the minimum duration of an awakening to allow for the encoding of some information into long term memory? What about the other components of the sleep microstructure?

Many previous results justified to investigate sleep microstructural components regarding dream recall. Sleep spindles and the alpha frequency during wakefulness have been related to memory abilities (Klimesch, 1997; Ulrich, 2016), the REMs of REM sleep to dream content (Dement and Kleitman, 1957; Roffwarg et al., 1962; Molinari and Foulkes, 1969), KCs to sleep stability (Bastien et al., 2000; Colrain, 2005; Halász, 2005) and muscles twitches may be considered as the physiological expression of oneiric behaviors thought to be an acting out of dreams (Wolpert, 1960; Sastre and Jouvet, 1979). Interestingly, our in-depth analysis of the sleep structure revealed no differences in the sleep microstructure of HR and LR for any of the parameters considered (i.e., arousals, sleep spindles, KCs, REMs and MTs). Since the number of stage shifts and the delta power in the first sleep cycle were also not significantly different between groups, these results leave awakenings as the only sleep parameter differentiating HR and LR sleep architectures. Our further analysis of these awakenings, notably their distribution in the sleep cycle, revealed that the higher dream recall frequency of HR could not be explained by the REM sleep hypothesis of dreaming (Dement and Kleitman, 1957; Ruby, 2011) since awakenings were not found to be more numerous or longer in REM sleep in HR as compared to LR (Figures 1A,D). Interestingly, we found that the frequency of the alpha predominant rhythm was slightly lower (of ~0.2 Hz) during ISW as compared to BSW. This is consistent with previous results that showed an increased power in the lower alpha range in the first minutes following an awakening as compared to the corresponding pre-sleep period (Ferrara et al., 2006). According to Klimesch (1997) which showed that the alpha frequency of good memory performers is about 1 Hz higher than those of bad performers, the decrease of alpha frequency in ISW could explain the difficulties in memory recall at awakening (recall of dreams or of awakenings during sleep). However alpha frequency was not different in HR and LR and can thus not explain their differences in dream recall.

What seems to be the best predictor of dream recall is the intra-sleep awakenings duration. During a night of sleep in the lab with sound presentation, in average, the awakenings of HR were twice longer than those of LR. The reanalysis of the data further showed that if the distribution of short (<1 min) and long awakenings (>1 min) is as expected in young healthy subjects (Goldenberg et al., 1981) i.e., with a great majority of short awakenings—the proportion of each category of awakenings was significantly different between the two groups. HR experienced a significantly higher proportion of long awakenings than LR, while LR experienced more short awakenings than HR. This result is interesting to discuss regarding those of a study which investigated the relationship between the duration of polysomnographic-defined awakenings and awakenings’ awareness (Campbell and Webb, 1981). The subjects’ task was to press a push-button whenever they gained awareness of being awake during sleep while polysomnographic data were acquired. They found that 84% of the unreported arousals/awakenings were shorter than 2 min (1.88 min). This result in addition to ours (awakenings but no arousals difference between HR and LR, average awakenings duration of 2 min vs. 1 min in HR vs. LR, more awakenings longer than 1 min in HR than in LR) suggest that awareness and/or memory of intra-sleep awakenings and dreams is dependent on the duration of intra-sleep awakenings and that the minimum duration for an awakening to allow for memory encoding is approximately 2 min. Given that according to available data the brain is not able to encode new information into explicit memory during sleep (Aarons, 1976), and that the brain is in a very different state during sleep and wakefulness, it seems not unrealistic that some time is needed to restore the encoding-in-memory ability of the brain at awakening.

Our results support but also extend and precise the arousal-retrieval model proposed by Koulack and Goodenough (1976). We found that intra-sleep awakenings duration is the only candidate among the numerous tested parameters assessing sleep macro and microstructure to explain dream recall, that the duration of awakenings rather than the frequency was the critical factor and that the required duration for an awakening to allow for memory encoding was approximately 2 min.

A limitation of this study is the smaller number of stimulus-related arousing reactions as compared to Bastuji et al. (2008) study with painful stimuli. For this reason we could not investigate AEPs in all the sleep stages. Second, automatic vs. visual detection of microstructural sleep parameters is a debated issue in the sleep community (e.g., Wallant et al., 2016). As a consequence even if no between groups difference have been observed, the figures (number of K complexes, spindles, MTs and REMs during REM sleep) have to be considered cautiously since they may have been different if the detection had been made visually. Thirdly, it should be kept in mind that our sleep measures does not reflect natural sleep since sounds were presented to participants during the whole night. That said, most of the computed sleep parameters were close or within the range of standard values, and it is therefore reasonable to assume that sleep quality was generally preserved despite the experimental setup and auditory stimuli. Finally, regarding the interpretation of the AEPs to arousing stimuli it should be kept in mind that the functional role of KCs regarding sleep depth is still unclear and debated, since it has been found that: (1) KCs do not change in the recovery night after sleep deprivation (Curcio et al., 2003); (2) KCs show a linear decline across the adult lifespan (Colrain et al., 2010); (3) they drop in patients with Alzheimer disease (Crowley et al., 2005); and (4) sensory stimulation increases their probability in the ascending slopes of the sleep cycles to a higher degree than in the descending ones (Colrain, 2005).

## Conclusion

We have shown that brain responses to auditory stimuli in N2 are larger when followed by a subsequent arousing reaction in the 5 s. Coherently with our previous results suggesting a greater brain reactivity in HR, HR demonstrated more arousing stimuli than LR during sleep. Finally, the reanalysis of our previous data (Eichenlaub et al., 2014a) highlighted that HR do not show any other sleep differences with LR apart from longer intra-sleep awakenings and, more precisely, more long and less short intra-sleep awakenings than LR. The results of our team lead us to propose the following mechanism leading to a better recall of dreams in HR than in LR. An increased activity in the temporo-parietal junction at rest in HR as compared to LR (Eichenlaub et al., 2014b) would lead to a greater brain reactivity to external stimuli (Eichenlaub et al., 2014a), i.e., larger brain responses to stimuli, which in turn would trigger more and longer arousing reactions during sleep. These intra-sleep awakenings would finally give more opportunities to the brain to restore his memory-encoding abilities and therefore to encode the dream in long term memory.

## Author Contributions

PMR, DM, J-BE designed the experiment and acquired data. RV, TL, J-BE, CB, KJ, DM and PMR participated in the data analysis. RV and PMR wrote the initial draft.

## Funding

This research was financially supported by a grant from the French National research Agency (Agence Nationale de la Recherche, ANR-07-JCJC-0095). This work was partly performed within the framework of the LABEX CORTEX (ANR-11-LABX-0042) of Université de Lyon, within the program ANR-11-IDEX-0007.

## Conflict of Interest Statement

CB has ownership and directorship in Physip S.A. Company. The other authors declare no conflict of interest.

## Abbreviations

AEP: Auditory evoked potential
AI: Arousal Index
BSW: Before sleep wakefulness
EEG: Electroencephalogram
EMG: Electromyogram
EOG: Electro-oculogram
ERP: Event related potential
HR: High dream recallers
Inds: Indeterminate
ISW: Intra sleep wakefulness
KCs: K-complexes
LR: Low dream recallers
MTs: Muscle twitches
Mvts: Movements
REMs: Rapid eyes movements
TST: Total Sleep Time.
