# Supplementary figures and images for: Increased Evoked Potentials to Arousing Auditory Stimuli during Sleep: Implication for the Understanding of Dream Recall
Source: Front Hum Neurosci. 2017 Mar 21;11:132. doi: 10.3389/fnhum.2017.00132 (PMC5360011; doi:10.3389/fnhum.2017.00132)

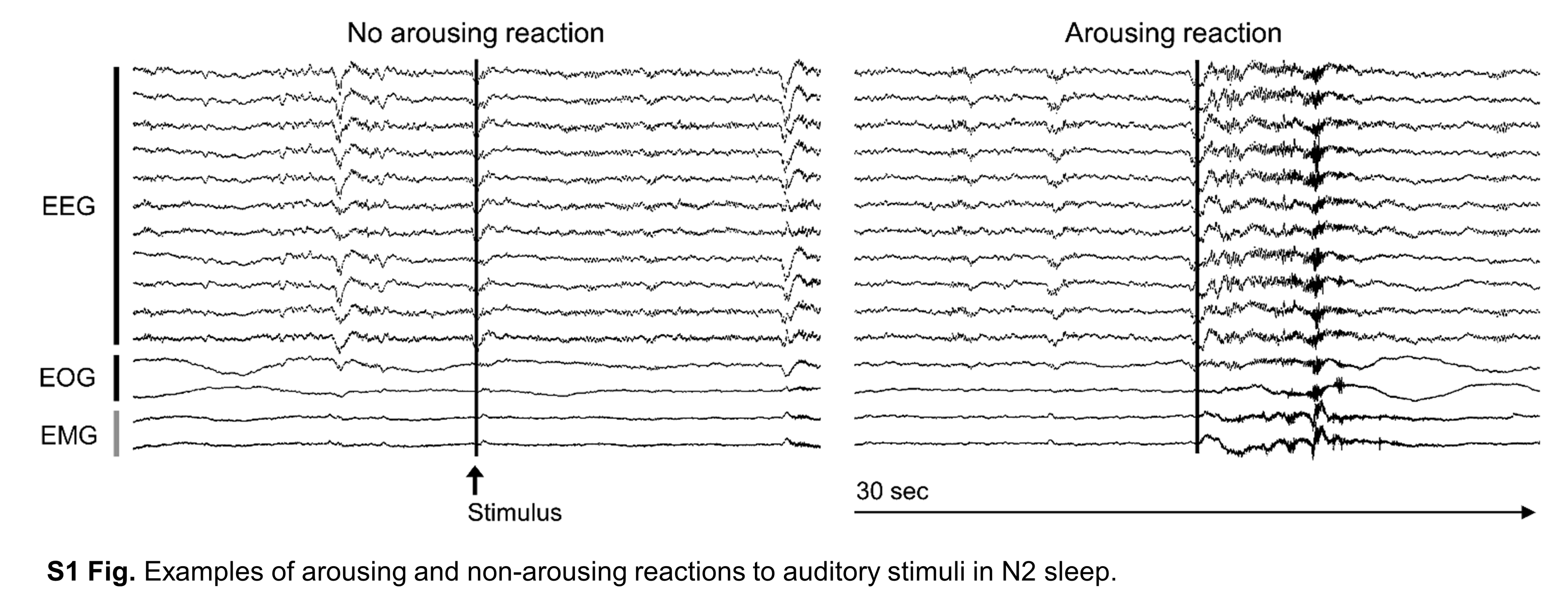

Supplement: Supplementary file 1 [file Image_1.tif]

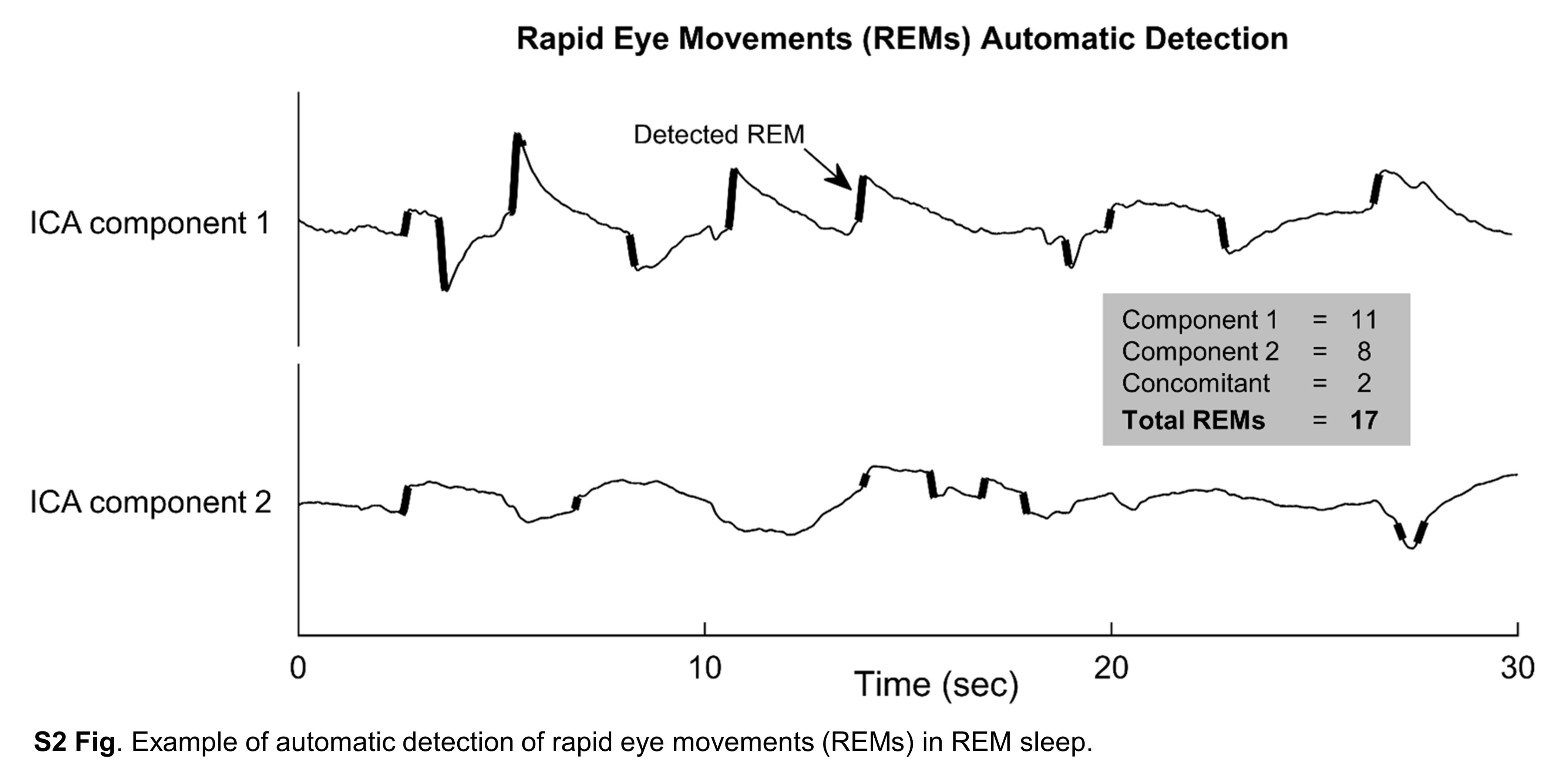

Supplement: Supplementary file 2 [file Image_2.tif]

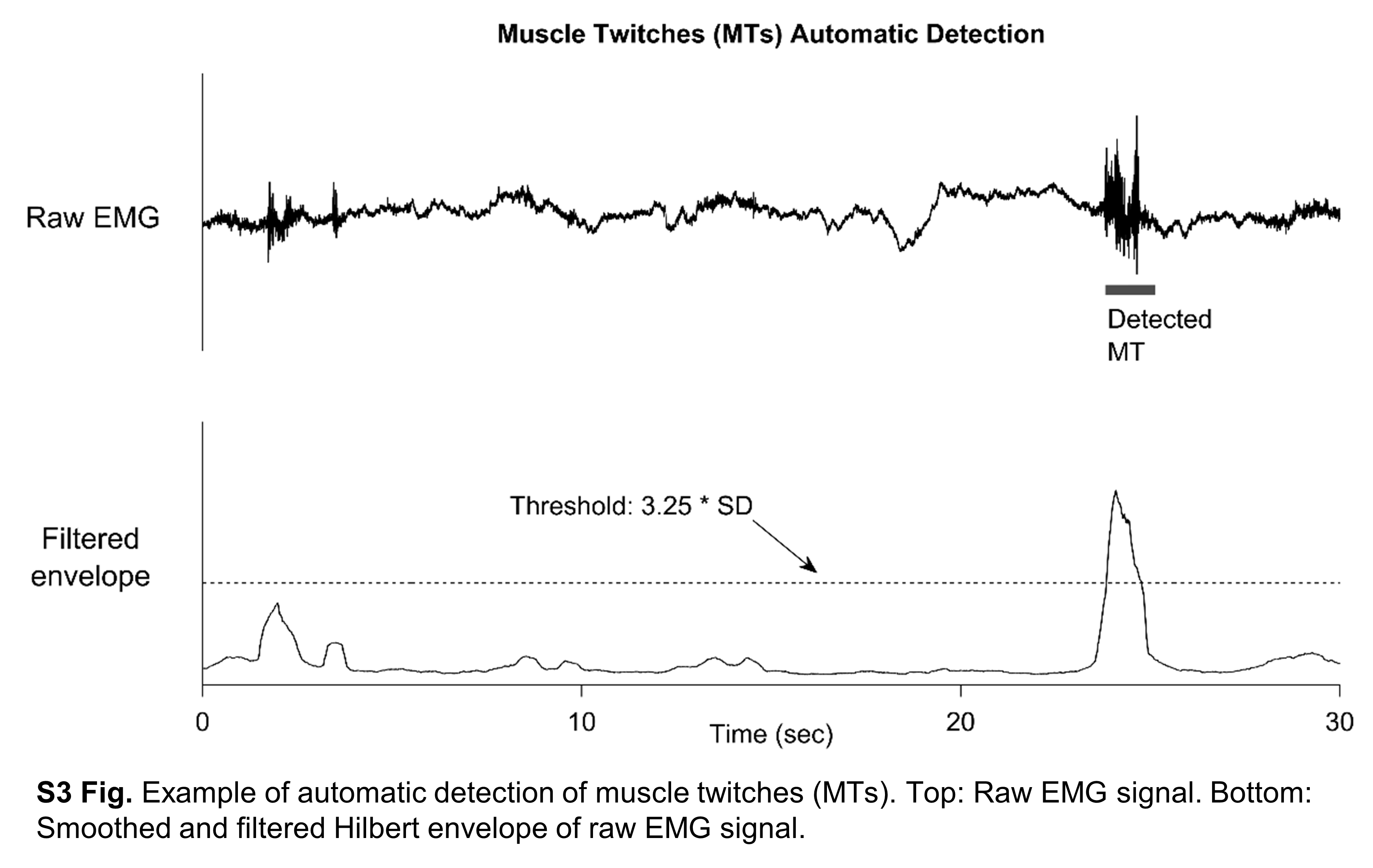

Supplement: Supplementary file 3 [file Image_3.tif]
