# Supplementary material for: Increased Evoked Potentials to Arousing Auditory Stimuli during Sleep: Implication for the Understanding of Dream Recall
Source: Front Hum Neurosci. 2017 Mar 21;11:132. doi: 10.3389/fnhum.2017.00132 (PMC5360011; doi:10.3389/fnhum.2017.00132)
Supplement: Supplementary file 4 [file Table_1.docx]

| S1 Table. Mean ± S.E.M of supplementary macrostructural parameters in High and Low dream recallers, with sleep onset defined as the first page of N1. | | | |
| --- | --- | --- | --- |
| Sleep parameters | **High-recallers** | **Low-recallers** | **Standard** |
| Macrostructural |  |  |  |
| Awakenings, no. | 19.7 ± 2.2 | 16.3 ± 4.1 | *9.6*^22^ |
| Awakenings, duration (min) | 1.9 ± 0.2 ** | 1.1 ± 0.1 | *1.4*^23^ |
| Awakenings Index, no. per hour | 3.6 ± 0.5 | 2.8 ± 0.7 | *4.2*^24^ |
| N1 | 26.5 ± 3.9 | 33.6 ± 4.9 |  |
| N2 | 3.4 ± 0.7 | 1.9 ± 0.8 |  |
| N3 | 1.0 ± 0.2 | 1.1 ± 0.3 |  |
| REM | 3.6 ± 1.5 | 1.0 ± 0.3 |  |
| Awakenings duration (%) |  |  |  |
| 0-1 min | 63.3 ± 3.5 ** | 80.3 ± 3.3 | *87*^11^ |
| 1-5 min | 28.9 ± 2.5 * | 18.1 ± 3.2 | *11*^11^ |
| 5-30 min | 7.8 ± 2.0 ** | 1.6 ± 0.7 | *3*^11^ |

Last column represents standard values. One-way and two-way ANOVA for independent samples (High-recallers versus Low-recallers) are presented: p<.05*, p<.01**.
